# A post-exercise infrared sauna session improves recovery of neuromuscular performance and muscle soreness after resistance exercise training

**DOI:** 10.5114/biolsport.2023.119289

**Published:** 2022-09-15

**Authors:** Essi K. Ahokas, Johanna K. Ihalainen, Helen G. Hanstock, Eero Savolainen, Heikki Kyröläinen

**Affiliations:** 1 Neuromuscular Research Center, Faculty of Sport and Health Sciences, University of Jyväskylä, Finland; 2 Swedish Winter Sports Research Centre, Department of Health Sciences, Mid Sweden University, Östersund, Sweden

**Keywords:** Infrared radiation, Recovery methods, Autonomic nervous system, Sauna, Neuromuscular performance

## Abstract

The aim of this study was to investigate effects of a single infrared sauna (IRS) session on post-exercise recovery of neuromuscular performance, autonomic nervous system function, subjective sleep quality, and muscle soreness. Male basketball players (n = 16) performed two trials consisting of a complex resistance exercise protocol (maximal strength with plyometrics), followed by either 20 min passive recovery (PAS) or IRS (temperature 43 ± 5°C), in a randomized crossover design, with trials separated by one week. Recovery of neuromuscular performance was assessed using 20 m maximal sprint, maximal countermovement-jump (CMJ), and isometric leg press tests, performed 14 hours after exercise. Heart rate (HR), heart rate variability (HRV), sleep diary, muscle soreness, and indirect muscle damage markers were measured pre and post exercise. The decrease in CMJ performance from pre- to post-exercise was attenuated after IRS compared to PAS (p < 0.01). The IRS session resulted in higher HR and lower root mean square of successive differences between normal heartbeats (RMSSD), and high and low frequency power, compared to PAS (p < 0.002). Post-exercise night-time HR and HRV did not differ following IRS vs. PAS. Muscle soreness was less severe, and perceived recovery was higher after IRS compared to PAS (p < 0.01). Post-exercise IRS attenuated the drop in explosive performance and decreased subjective muscle soreness after resistance training, which may enhance mood, readiness, and physical performance of an athlete. A single IRS session had no detrimental effects on recovery of the autonomic nervous system.

## INTRODUCTION

Infrared radiation (IR) is a subdivision of the electromagnetic spectrum with wavelengths between 750 nm –1 mm [1]. The IR-region is often divided into IR-A (0.78–1.4 μm), IR-B (1.4–3 μm), and IR-C (3 μm–1 mm), the latter of which is also termed far-infrared radiation (FIR) [1]. IR radiates energy in the form of heat [1]. Thermal radiation has effectively been used to alleviate certain diseases and discomforts, such as musculoskeletal disorders, and IR-emitting saunas (IRS) and heat lamps are gaining popularity [1]. In line with these technological developments, the use of IR for relaxation and recovery is becoming more widespread. An IRS heats the air to approximately 40–60°C and radiates heat at wavelengths between 1–12 μm [2, 3]. The manufacturers of IRS advertise numerous therapeutic effects, including weight loss, improved cardiovascular health, reduced pain, and fatigue. However, the scientific evidence to support these claims is limited due to low number of studies and conflicting results [2].

Research evidence is also lacking on whether IRS can influence recovery of physical performance. A post-exercise IRS session has been found to improve recovery of countermovement jump (CMJ) performance [3]. Exposure to a IR-lamp has been found to reduce muscle soreness but had no effect on creatine kinase (CK) activity, an indirect biomarker of exercise-induced muscle damage [4]. The beneficial effects of IR during recovery may be attributed to increased peripheral blood flow due to vasodilatation [5], which could accelerate the clearance of edema, limit inflammation and perceived pain, and improve muscle repair [4]. As IR heat penetrates deeper under the skin than warm air, it may transmit heat to muscles, blood vessels, and nerves more effectively [2, 6], which may attenuate tissue temperature loss, facilitate muscle circulation and metabolism, and reduce peripheral nerve excitability [7].

Heart rate variability (HRV) is a tool for monitoring autonomic nervous system balance [8, 9]. A post-exercise IRS session has been found to decrease HRV and increase heart rate (HR) indicating prolonged sympathetic activity after exercise [10]. On the other hand, a IRS session without prior exercise did not affect HRV and HR [10]. Furthermore, repeated use of IRS has been found to improve sleep quality in patients with chronic pain [11]. However, the effects of post-exercise IRS on subsequent nocturnal HRV and sleep quality have not been studied. It would be important to verify that post-exercise IRS does not place additional stress on autonomic function after exercise. Hormonal concentrations in blood samples can provide additional information about the physiology underlying the recovery process by indicating stress responses and anabolic-catabolic balance [12].

The aim of the present study was to investigate effects of a single post-exercise full-spectrum IRS session on recovery of neuromuscular performance, autonomic nervous system balance, subjective sleep quality, muscle soreness, biomarkers of muscle damage, and hormonal responses. We hypothesized that IRS after resistance training may enhance recovery of neuromuscular performance, increase nocturnal HRV, and attenuate muscle soreness, as well as biomarkers of muscle damage.

## MATERIALS AND METHODS

### Participants

Sixteen healthy and injury-free male basketball players (mean ± SD: age 18.9 ± 2.3 years, height 189 ± 8 cm, body mass 83.4 ± 11.1 kg, body fat 13.3 ± 5.7%) provided written informed consent to take part in the study. For participants who were under the age of 18 years, written consent from a guardian was also obtained. Participants represented two different teams from same club: the first team (n = 7), playing in the second highest league in Finland, and the second team (n = 9), playing in the third highest league in Finland. Ethical approval for the study was obtained from the University of Jyväskylä Ethical Committee. The study was conducted in accordance with the Declaration of Helsinki.

### Research Design

**FIG. 1:**
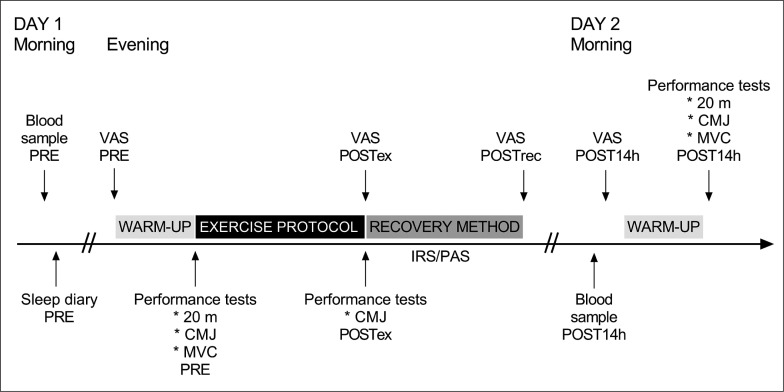
Schematic view of study design. Participants completed a standardized exercise protocol followed by a recovery protocol, involving sitting in infrared sauna (IRS) or in room temperature (PAS, passive recovery). Follow-up measurements were obtained the morning after exercise protocol. PRE = before the exercise protocol; POSTex = after the exercise protocol; POSTrec = after the recovery method; POST14h = after 14 hours recovery; 20 m = 20 m sprint; CMJ = countermovement jump; MVC = isometric leg press; VAS = visual analog scale by muscle soreness.

Participants performed two experimental trials that each took place over two days, separated by one week. First, participants performed a resistance exercise protocol. IRS (43 ± 5°C, 20 min) and passive recovery (PAS, room temperature, 20 min) were used after exercise in a randomized order. A schematic representation of the protocol is provided in Figure 1.

Participants performed 20 m maximal sprint, maximal counter-movement-jump (CMJ), and isometric leg press tests before (pre) and 14 h after exercise (post14h). The CMJ test was also performed immediately after exercise (postEX). We collected blood samples in the morning (pre) and at post14h. Participants completed sleep diaries in the mornings before and after the exercise protocol, and muscle soreness questionnaires (Visual Analog Scale, VAS) at pre, postEX, immediately after the recovery intervention (postREC), and post14h. HRV was assessed during the nights before and after exercise, as well as during IRS and PAS.

Fluid intake was controlled during the experimental trials. Participants were provided with 0.5 l water during the exercise protocol and 0.5 l water during IRS or PAS. Participants consumed a recovery beverage (8 g carbohydrates and 28 g protein, Teho Sport, Olvi Oyj, Iisalmi, Finland) before IRS and PAS. Furthermore, participants completed food diaries during the first recovery period to track nutritional intake during the first recovery period and ensure replication of it during the second recovery period. The use of nonsteroidal anti-inflammatory drugs and other recovery methods was prohibited.

### Procedures

#### Exercise protocol

Participants performed a complex resistance exercise protocol (total duration 60 min), which participants were accustomed to performing, as follows:

1. Back squat 3 × 3 + CMJ 3 × 3 (90–95% 1RM; 1 min rest between squat and jump, 3 min rest between sets)
2. Nordic hamstring curl 3 × 5 + standing long jump 3 × 3 (maximal effort; 1 min rest between curl and jump, 3 min rest between sets)
3. Leg press 3 × 3 + box jump (90–95% 1RM; 1 min rest between leg press and jump, 3 min rest between sets)

During the first trial, participants performed the exercises in a randomised order and completed warm-up sets as they felt was appropriate. The exercise order, weights, and warm-up sets were then replicated for each participant during the second trial. During the exercise protocol, participants reported their Rating of Perceived Exertion (RPE, scale 1–10) after the last set of each exercise, and the mean RPE value was calculated.

#### Performance tests

To assess acute fatigue, a CMJ-test was performed 3 minutes after completion of the exercise protocol. Furthermore, pre-, and post-performance tests included a CMJ, 20 m maximal sprint, and isometric leg press. The CMJ flight time, determined using photocells (University of Jyväskylä, Finland), was used as a measure of maximal muscle power output. Participants kept their hands on their hips and jumped as high as possible, with three attempts to achieve their best performance (1 min rest between attempts). A self-determined range of motion was permitted. In the 20 m sprint test, we used photocells (University of Jyväskylä, Finland) to obtain sprint times. Participants had three attempts to achieve their best sprint performance, with 3 min rest between attempts. We used an isometric leg press device (University of Jyväskylä, Finland) to measure maximal voluntary contraction, performed with a knee angle of 110°, which has been reported previously [13]. Participants had three attempts to achieve their best performance with 1 min rest between attempts.

#### Infrared sauna session

Participants sat for 20 min in an infrared sauna (VitaMy, Sentiotec GmbH, Vöcklabruck, Austria), or engaged in passive recovery by sitting in room temperature for 20 min. The sauna had two seats with IR-emitting panels (radiation: IR-A 24%, IR-B 55%, IR-C 24%) at the front (distance: about 55 cm from the chest of the participant) and back (distance: 7–15 cm from the back of the participant). Temperature of the sauna was set to 50°C, but the measured air temperature was 43 ± 5°C and relative humidity 21 ± 1% in the sauna.

#### Heart rate and heart rate variability monitoring

A wearable sensor (Bodyguard2, BG2, Firstbeat Technologies Ltd, Jyväskylä, Finland) was used to record HR and HRV. The sensor uses two spot electrodes, which were placed under the right collarbone and on the left rib cage. Participants attached the monitor before going to bed on the day before the exercise protocol and wore the monitor continuously until the morning after the exercise protocol. The monitor records an electrocardiogram and converts the signal into R-R intervals using an algorithm with an accuracy of 1 ms. The BG2 sensor has been found to be accurate and reliable for HRV analysis [14]. We transferred data from the sensor to the validated [15] Kubios software (Kubios Oy, Kuopio, Finland) for further analysis. Average HR, the root mean square of successive differences between normal heartbeats (RMSSD), power of high frequency (HF-power) and low frequency (LF-power) heart rate variability, and ratio of low and high frequency heart rate variability (LF/HF-ratio) were analyzed (Table 1).

**TABLE 1:** Heart rate variability variables analyzed.

Variable	Explanation
HR (l/min)	Average heart rate
RMSSD (ms)	The root mean square of successive differences between normal heartbeats
HF-power (ms^2^)	Average power of high frequency heart rate variability (0.15–0.40 Hz)
LF-power (ms^2^)	Average power of low frequency heart rate variability (0.04–0.15 Hz)
LF/HF-ratio	Ratio of low and high frequency heart rate variability (LF-power/HF-power)

HRV data were analyzed from each night over a four-hour period, commencing 30 min after bedtime. Furthermore, HRV data were analyzed over 16 min during the recovery sessions. The raw HRV data required little artifact correction; the average percentage of corrected beats was 1.3% (117 beats) during the night and 0.5% (6 beats) during the recovery sessions.

#### Sleep diaries and muscle soreness

Sleep diaries were used to evaluate participants’ bedtimes, as well as the duration and quality (on a scale of 1–5) of sleep. Furthermore, participants reported perceived recovery (on a scale of 1–5). We used a VAS scale to evaluate muscle soreness; reported on a 12 cm line with “no pain” at one end (0 cm) and “the worst possible pain” at the other end (12 cm).

#### Collection and analysis of blood samples

Blood samples were collected to analyze serum hormone and myoglobin concentrations and CK activity in the mornings (7:30–9:15 a.m.), after 10 h of fasting. Blood samples were taken from the antecubital vein into serum tubes (Vacuette, Greiner Bio One International GmbH, Kremsmünster, Austria). Whole blood was centrifuged at 3600 rpm (Megafuge 1.0 R, Heraeus, Germany) for 10 min. After separation of serum, samples were frozen at -80°C until analysis. To assess anabolic versus catabolic status and muscle damage, we measured serum cortisol, total testosterone, sex hormone binding globulin (SHBG), growth hormone, and myoglobin levels with chemiluminescence immunoassays (IMMULITE 2000 XPi, Siemens Healthcare Diagnostics, UK) and hormone-specific immunoassay kits (Siemens, New York City, USA). CK activity was assessed with a photometric test (Indiko Plus, Thermo Fisher, Massachusetts, USA). The interassay coefficients of variation were 5.1% for cortisol, 4.1% for growth hormone, 2.1% for myoglobin, 6.3% for SHBG, and 13.1% for testosterone.

Serum free testosterone was calculated using Anderson’s formula: Free testosterone (pmol/l) = Total testosterone (nmol/l) × (2.28–1.38 × log(SHBG (nmol/l) /10)) × 10 [16]. Hemoglobin and hematocrit were determined with an automated hematological analyzer (Sysmex XP300, Sysmex Corporation, Kobe, Japan), and plasma volume change from pre was determined from hemoglobin and hematocrit concentrations using the equation by Dill and Costill [17]. The plasma volume corrected values were used in analyses.

#### Data processing

One participant (A) could not participate in all the CMJ measurements, two participants (A, B) could not complete all 20 m sprints, and three participants (A, C, D) could not complete all the isometric leg press measurements. Absences were due to personal scheduling problems (A) and soft tissue pain/discomfort (B, C, D). The physical performance results from one participant (D) deviated above mean + 2SD and were not included in the analyses. Thus, the number of participants included in the analysis of physical performance were: 20 m sprint: n = 13; CMJ: n = 14; and isometric leg press: n = 12.

HRV data from four participants were not obtained for the entire four-hour periods and in some cases (6 participants) the period began later than 30 min after bedtime, because of missing data or longer sleep onset latency reported in the sleep diary. In these cases, participants’ data were analyzed in the same way for both experimental conditions. A total of 5 participants had missing data from one or more nights. Thus, nocturnal HRV data were analyzed from 11 participants. Furthermore, HRV data during the recovery methods was not obtained from 5 participants, thus 11 participants were included in the analysis. One participant ate before collection of a fasted blood sample. His results were not taken into account in the statistical analyses of the blood samples.

#### Statistical Analyses

Data were analyzed using IBM SPSS Statistics 26 (IBM Corp. Armonk, NY, USA). Two-way repeated measures analysis of variance (ANOVA) followed by Bonferroni post hoc tests, as well as Friedman and Wilcoxon tests were used in the statistical analysis. Paired t-tests were used to analyze absolute or relative (physical performance) changes (pre-post) between the recovery methods. Data are presented as mean ± SD. Statistical significance was set at p < 0.05. Effect sizes were calculated (ES; Cohen’s d) to determine meaningful differences. CK and myoglobin were analyzed as natural logarithms (ln). The change between the pre- and post-measurements was calculated as: lnChange = ln(post/pre).

## RESULTS

### Exercise effort and acute fatigue

The relative decrease in CMJ performance from pre- to post-exercise did not differ (p = 0.158) between IRS (-6.0 ± 4.6%) and PAS (-7.9 ± 3.7%). Neither did mean RPE differ between the exercise protocols that preceded IRS and PAS (IRS: 8.1 ± 0.9; PAS: 8.3 ± 0.8, p = 0.248).

### Neuromuscular performance

IRS attenuated the relative reduction in CMJ performance from pre- to post14h (p = 0.009, ES = 0.76) compared to PAS (Table 2). However, there was no difference in CMJ performance at post14h between IRS and PAS (p = 0.129, ES = 0.65). The post-exercise decrease in CMJ height was statistically significant in the PAS trial (p = 0.002, ES = 1.28), but not the IRS trial (p = 1.000).

**TABLE 2:** Mean (SD) results and relative changes from pre- to post-measurement for countermovement jump, 20 m sprint, and isometric leg press in the infrared sauna and passive recovery.

	IRS	PAS	%Δ IRS	%Δ PAS
CMJ (cm)					
Pre	41.5 (5.5)	41.8 (5.6)		
Post 14 h	40.9 (4.8)	39.8 (5.7)^##^	-1.1 (4.5)^**^	-5.0 (3.8)

20 m sprint (s)					
Pre	2.95 (0.11)	2.96 (0.11)		
Post 14 h	3.03 (0.12)^###^	3.04 (0.10) ^###^	2.5 (1.4)	2.7 (1.2)

Isometric leg press (kg)					
Pre	673 (221)	700 (218)		
Post 14 h	678 (240)	692 (234)	0.0 (10.0)	-2.2 (6.7)

Abbreviation: CMJ = countermovement jump; IRS = infrared sauna; PAS = passive recovery; %Δ = percentage changes from pre- to post-measurement.

** Indicates difference compared to passive recovery (p < 0.01).

### Indicates difference from pre-value (p < 0.001).

## Indicates difference from pre-value (p < 0.01).

The relative changes in sprint time and isometric leg press performance did not differ between trials from pre- to post14h (p = 0.631 and p = 0.545, respectively; Table 2). Furthermore, there were no differences in 20 m sprint and isometric leg press performance 14 h after recovery with IRS or PAS (p = 1.000). However, there was an increase in 20 m sprint time at post14h in both trials (p < 0.001).

### Heart rate and heart rate variability

HR and LF/HF-ratio were higher (p < 0.001, ES = 2.39; p = 0.002, ES = 1.23), and RMSSD, HF-power and LF-power were lower (p < 0.001, ES = 2.08; p = 0.001; ES = 1.50; p = 0.002, ES = 1.24) during IRS compared to PAS recovery (Table 3). No differences in the changes from night before to night after values were observed between the recovery protocols. There was no significant time or treatment effect on nocturnal HR and HRV (Table 3).

**TABLE 3:** Mean (SD) heart rate and heart rate variability variables in the infrared sauna and passive during recovery conditions, during the night before and after the exercise protocol, and change from the night before to the night after values.

	IRS	PAS
**During recovery method**		
RMSSD (ms)	26.7 (8.9)^***^	53.4 (15.0)
HR (1/min)	85 (6)^***^	72 (5)
HF-power (ms^2^)	295 (180)^**^	1027 (545)
LF-power (ms^2^)	1214 (623)^**^	2649 (1600)
LF/HF-ratio	4.83 (2.13)^**^	2.82 (1.27)

**Night before**		
RMSSD (ms)	84.7 (41.2)	77.7 (32.2)
HR (1/min)	53 (7)	52 (7)
HF-power (ms^2^)	2372 (1768)	1947 (1333)
LF-power (ms^2^)	2813 (1929)	2296 (1151)
LF/HF-ratio	1.34 (0.57)	1.41 (0.51)

**Night after**		
RMSSD (ms)	77.2 (37.4)	76.6 (36.4)
HR (1/min)	53 (5)	52 (5)
HF-power (ms^2^)	2021 (1449)	1940 (1499)
LF-power (ms^2^)	2472 (1723)	2294 (1199)
LF/HF-ratio	1.40 (0.51)	1.48 (0.65)

**Δ**		
RMSSD (ms)	-7.5 (8.7)	-1.0 (15.8)
HR (1/min)	-0.1 (3.1)	-0.4 (3.2)
HF-power (ms^2^)	-351 (477)	-6 (1148)
LF-power (ms^2^)	-342 (513)	-2 (530)
LF/HF-ratio	0.1 (0.3)	0.1 (0.6)

Abbreviation: IRS = infrared sauna; PAS = passive recovery; Δ = changes from night before to night after measurement; RMSSD = root mean square of successive differences; HR = heart rate; HF-power = average power of high frequency heart rate variability; LF-power = average power of low frequency heart rate variability; LF/HF-ratio = ratio of low and high frequency heart rate variability.

*** Indicates difference compared to passive recovery (p < 0.001).

** Indicates difference compared to passive recovery (p < 0.01).

### Hormonal responses

The change in cortisol from pre- to post14h did not differ between IRS and PAS (p = 0.306; Table 4). However, higher cortisol values (p = 0.005, ES = 0.518) were found at post14h following IRS compared to PAS. Furthermore, there was a decrease in cortisol values from pre to post14h after PAS (p = 0.001, ES = 0.61), but not after IRS (p = 0.256). There were no differences in other hormone values (Table 4).

**TABLE 4:** Mean (SD) hormonal values and changes from pre- to post-measurement in the infrared sauna and passive recovery conditions.

	IRS	PAS	Δ IRS	Δ PAS
Cortisol (nmol/L)				
Pre	445 (98)	432 (59)		
Post 14 h	423 (58)^**^	390 (57)^##^	-21 (64)	-42 (39)

Total testosterone (nmol/L)				
Pre	17.1 (4.2)	17.3 (3.7)		
Post 14 h	16.8 (4.7)	15.9 (2.9)	-0.2 (2.5)	-1.4 (2.4)

Free testosterone (pmol/L)				
Pre	265 (43)	267 (42)		
Post 14 h	261 (51)	252 (38)	-3 (33)	-15 (32)

Growth hormone (ug/L)				
Pre	0.256 (0.217)	0.118 (0.102)		
Post 14 h	0.231 (0.279)	0.105 (0.077)	-0.025 (0.344)	-0.013 (0.126)

Abbreviation: IRS = infrared sauna; PAS = passive recovery; Δ = changes from pre- to post-measurement.

** Indicates difference compared to passive recovery (p < 0.01).

## Indicates difference from pre-value (p < 0.01).

### Muscle soreness, creatine kinase, and myoglobin

The change in muscle soreness from pre- to post14h-values was smaller after IRS compared to PAS (p = 0.032, ES = 0.58; Table 5). Furthermore, muscle soreness was significantly affected by the recovery protocols and varied over the time course of recovery. Post-exercise muscle soreness was higher following both protocols compared to pre-values (IRS: p = 0.002, ES = 0.54; PAS: p = 0.001, ES = 0.56). Absolute muscle soreness scores were lower after IRS compared to PAS at the postREC- (p = 0.003, ES = 0.53) and post14h-measurements (p = 0.005, ES = 0.50).

**TABLE 5:** Mean (SD) muscle soreness and changes from pre- to post-measurement in the infrared sauna and passive recovery conditions.

	IRS	PAS	Δ IRS	Δ PAS
Pre	2.0 (1.7)	3.1 (2.3)		
Post exercise	4.7 (2.2)^##^	5.8 (3.0)^##^	2.8 (2.6)	2.8 (2.5)
Post recovery method	3.0 (2.0)^**, §§^	5.3 (2.8)^##^	1.1 (2.7)	2.2 (2.3)
Post 14 h	2.9 (2.0)^**, §§^	5.2 (3.0) ^##^	0.9 (2.3)^*^	2.1 (1.8)
Post recovery method	3.0 (2.0)^**, §§^	5.3 (2.8) ^##^	1.1 (2.7)	2.2 (2.3)
Post 14 h	2.9 (2.0)^**, §§^	5.2 (3.0) ^##^	0.9 (2.3)^*^	2.1 (1.8)

Abbreviation: IRS = infrared sauna; PAS = passive recovery; Δ = changes from pre- to post-measurement.

** Indicates difference compared to passive recovery (p < 0.01).

* Indicates difference compared to passive recovery (p < 0.05).

## Indicates difference from pre-value (p < 0.01).

§§ Indicates difference from post exercise value (p < 0.01).

**FIG. 2:**
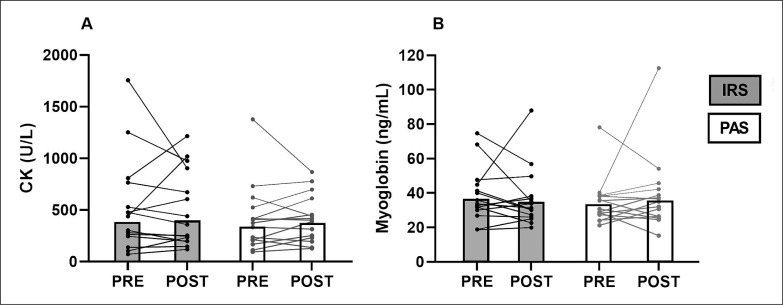
Geometric means and individual values of CK (A) and myoglobin (B). IRS = infrared sauna method; PAS = passive recovery; Pre = before exercise protocol; Post = 14 h after exercise protocol.

There were no differences in CK and myoglobin between IRS and PAS in either the change from the pre- to post14h-values (CK: p = 0.640; Myoglobin: p = 0.446); or the absolute values (CK: p = 1.000; Myoglobin: p = 0.955; Figure 2).

### Sleep quantity, sleep quality, and perceived recovery

**FIG. 3:**
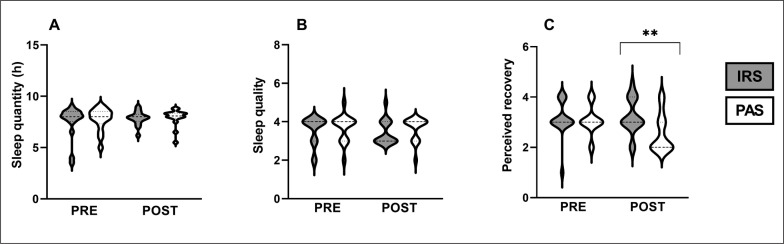
Violin plots of sleep quantity (A), sleep quality (B), and perceived recovery (C). ** Statistically significant (p < 0.01) difference between far-infrared sauna and passive recovery. IRS = infrared sauna method; PAS = passive recovery; Pre = before exercise protocol; Post = 14 h after exercise protocol.

Sleep duration and quality did not differ between IRS and PAS (duration: p = 0.377, Figure 3A; quality: p = 0.632, Figure 3B), or between the two nights (duration: IRS: p = 0.727, PAS: p = 0.201; quality: IRS: p = 0.480; PAS: p = 0.705). However, the change in perceived recovery from pre to post14h was greater in IRS (0.1 ± 0.7) compared to PAS (-0.5 ± 0.7; p = 0.009, ES = 0.47). Perceived recovery the morning after exercise was also higher at post14h in IRS (3.2 ± 0.7) compared to PAS (2.6 ± 0.8; p = 0.007, ES = 0.48, Figure 3C). Furthermore, following PAS, the perceived recovery at post14h was lower compared to pre (p = 0.027, ES = 0.392). This reduction was not noticed after IRS (p = 0.527).

## DISCUSSION

The main finding of this study was that an IRS session improves recovery of power capacity in the lower extremities, assessed by CMJ, and attenuates muscle soreness, as we hypothesized. Contrary to our hypothesis, IRS had no effect on nocturnal HRV and subjective sleep quality.

In the present study, the deterioration of CMJ performance during recovery was attenuated by IRS compared to PAS. IRS during recovery has previously been found to improve CMJ performance after a single endurance training session, but not after a strength training session [3]. In that study, however, participants were not athletes and performed a hypertrophic strength training session [3]. Furthermore, recovery of power capacity improved after using a farinfrared ‘sleeping’ bag four times during a five-day training period [6]. Because relatively little research has been conducted on IRS and recovery of physical performance, it may also be important to compare our findings with studies that have examined recovery after traditional sauna bathing. Traditional saunas heat the air to approximately 70–100°C, which then heats the occupant primarily by convection [2, 3]. In other words, heat is transferred by the movement of the heated air to the occupant. In contrast, IRS radiates heat [2, 3]. It is believed that radiated infrared heat penetrates deeper into human tissues, such as muscle tissue, than convection of heated air [2]. A traditional sauna bathing session with and without prior exercise was found to decrease strength capacities for 24 h during recovery [18]. In addition, taking a traditional sauna after swimming was found to impair swimming performance the following morning [19]. These negative effects have not been found with IRS [3, 6].

Previous studies have speculated that the positive effect of IR on neuromuscular performance can be attributed to deeper heat penetration, that upon reaching the neuromuscular system [3] elicits positive effects on the function of fast-twitch muscle fibers [6], located predominantly in the superficial layers of the muscle [20]. Furthermore, it has been speculated that IR could alter cell membrane potentials [1], but this has not been studied with IRS while the similar radiators and wavelengths has been used. However, we found no differences between the recovery protocols in 20 m sprint and isometric leg press performance, in line with previous studies [3, 6]. Others have observed improved CMJ performance following recovery interventions, but no effects on isometric maximal strength tests [21, 22]. This discrepancy in results from different performance tests may be attributed to heterogenous demands on neuromuscular mechanisms during the stretch-shortening cycle [22] or enhanced recovery of fasttwitch muscle fibers [21]. Sprint performance predominantly depends on muscle strength capacity and neuromuscular coordination [23], so it may not be sufficiently sensitive to minor differences in physiological recovery.

During the IRS session, we found decreased parasympathetic activity compared to passive recovery, indicating that the IRS session may have increased stress in the body. However, nocturnal HR and HRV-values did not differ between recovery protocols. Thus, the stress caused by IRS is likely short-lived. In a previous study, post-exercise IRS was found to acutely decrease RMSSD and increase HR compared to pre-exercise values, but RMSSD-values were higher after IRS compared to after exercise [10]. Because of missing data, the number of participants in HRV analyses were low, and the effect of IRS on HRV should be studied with larger number of participants. Nonetheless, it appears that a post-exercise IRS does not affect restoration of autonomic modulation during sleep, even after an evening training session. Moreover, there were no differences in subjectively assessed sleep quality and quantity in this study. However, after IRS, participants reported improved perceived recovery the following morning. Thus, it appears that IRS may have positive effects on subjective recovery, but the effect of IRS on post-exercise sleep quality is not clear.

We found higher cortisol concentrations after post IRS compared to passive recovery the following morning. Nonetheless, the change in cortisol from pre- to post-intervention was not statistically significant between protocols, and the post14h concentration was not higher than pre-concentration after IRS. There were neither changes over time nor differences between IRS and passive recovery in other hormone variables. Similarly, in one previous study, it was found that repeated use of an FIR ‘sleeping’ bag during recovery had no effect on cortisol and testosterone concentrations [6]. Contrary, growth hormone has been shown to increase following a traditional sauna session after resistance exercise, while testosterone was elevated 24 hours after a sauna session [18]. Because hormonal changes were observed at different time points and with different exercise protocols, it cannot be concluded from the present study whether traditional sauna and IRS may exert different effects on hormonal responses. In the present study, IRS did not appear to influence hormonal balance during the follow-up period. However, it would be important to study the effect of IRS on hormonal responses in the first few minutes and hours after exercise, because testosterone [24, 25], cortisol, and growth hormone levels [26] have been found to increase immediately after exercise and return to or below baseline within one hour.

IRS reduced muscle soreness compared to pre-exercise and passive recovery in the present study. In previous study, the use of a IR-lamp has been found to decrease perceived pain after 48 hours recovery compared to passive recovery [4]. Furthermore, meta-analytical data show that the use of post-exercise heat therapy after exercise can relieve pain for more than 24 hours [7]. It has been speculated that post-exercise heat therapy may reduce muscle tissue temperature loss, thereby facilitating circulation and energy metabolism in the muscle, which reduces accumulation of inflammatory cytokines and metabolic waste in muscles [7]. In addition, heat may decrease the excitability of peripheral nerves, and a combination of these factors may contribute to pain relief [7]. Nevertheless, markers of muscle damage did not differ between IRS and passive recovery. CK activity and myoglobin values were already elevated at the start of each trial, possibly because participants performed the trials during the competitive or preparatory phase of their season, which may have influenced the results of CK activity and myoglobin concentration. On the other hand, in previous studies CK activity did not decrease after the use of IR-lamps [4], IR-clothes [27, 28], and a IR-bag [6] compared to control conditions. Thus, based on this study and previous studies, it seems that IR might not affect CK activity and myoglobin concentration.

It should be noted that because of the nature of IRS, it is not possible to provide a placebo condition within such experimental trials. Therefore, it is possible that participants expected a positive outcome from IRS, which may have influenced the results of the subjectively-assessed variables. The improved perception of recovery and muscle soreness may also lead to an improvement in athletes’ mood and readiness, which in turn may enhance physical performance [29]. On the other hand, the effectiveness of IRS could be investigated by including another heat condition as a placebo in the future. It should be considered that the participants in this study were competitive athletes who were familiar with the exercise modalities employed during the exercise session. Therefore, generalization of the results to athletes from other sports is more applicable, although only for short recovery periods.

## CONCLUSIONS

A single post-exercise IRS session improved recovery of explosive strength capacities and decreased subjective muscle soreness one day after resistance exercise. In addition, a single IRS session had no detrimental effects on acute recovery of the autonomic nervous system or sleep quality, but conversely increased subjective perceived recovery the following morning. An adequate balance between training and competition load and recovery is essential for athletes to achieve continuous high-level performance. The improved recovery could indicate a better readiness to train and compete. Thus, IRS could be a practically useful recovery method for athletes since it is safe and relatively easy to integrate into athletes’ training routines. Further studies should investigate the mechanisms that may underpin the performance enhancement and reduced muscle soreness following post-exercise IRS.
